# Adsorption and desorption of heavy metals by the sewage sludge and biochar-amended soil

**DOI:** 10.1007/s10653-017-0036-1

**Published:** 2017-11-07

**Authors:** Aleksandra Bogusz, Patryk Oleszczuk, Ryszard Dobrowolski

**Affiliations:** 1 Department of Environmental Chemistry, Faculty of Chemistry, Maria Sklodowska-Curie University, pl. M. Curie-Skłodowskiej 3, 20-031 Lublin, Poland; 2 Department of Analytical Chemistry, Faculty of Chemistry, Maria Skłodowska-Curie University, Maria Curie-Skłodowska Square 3, 20-031 Lublin, Poland

**Keywords:** PTEs, Biochar, Sewage sludge, Adsorption, Desorption, Soil

## Abstract

The goal of the study was to evaluate the application of biochar (BC) to the sewage sludge (SL) on the adsorption and desorption capacity of Cd(II), Cu(II), Ni(II) and Zn(II). The effect of biochar contribution in the sewage sludge (2.5, 5 and 10%) was investigated. The isotherms data were fitted to the Langmiur (LM), Freundlich (FM) and Temkin (TM) models. The best fitting for kinetic study was obtained for the pseudo-second-order equation. The best fitting of the experimental data was observed for the LM in the case of SL and BC, and for the FM in the case of SL- and SL/BC-amended soil. SL was characterized by even four-order higher sorption capacity than BC. The addition of the BC to the SL and next to the soil increased the adsorption capacity of the soil and the SL-amended soil. In the case of all investigated potentially toxic elements (PTEs), the highest adsorption capacity was achieved for SL-amended soil in comparison with the control soil. In the case of other experimental variants, the adsorption capacity of metal ions was as follows: 2.5% BC > 5.0% BC > 10% BC. The negative correlation between hydrated radius of metal ions and the kinetics of sorption was observed. However, the desorption of PTEs from BC/SL-amended soil was significantly lower than for SL-amended soil (except of Cd) and non-amended soil. It can be concluded that the addition of the biochar enhanced the immobilization of PTEs and reduced their bioavailability and mobility in the soil amended by the sewage sludge.

**Electronic supplementary material:**

The online version of this article (doi:10.1007/s10653-017-0036-1) contains supplementary material, which is available to authorized users.

## Introduction

Sewage sludge (SL) as a product of the wastewater treatment can be considered as one of the most difficult material to manage. This difficulty is connected not only with constantly raising amount of the produced sewage sludge, but also with sewage sludge composition, especially contaminants content. Sewage sludge contains a lot of different contaminants, including potentially toxic elements (PTEs), polycyclic aromatic hydrocarbons (PAHs), personal care products (PCPs) as well as pathogens (Devi and Saroha 2017). Special group of contaminants are PTEs because they are highly dangerous for the environment, non-biodegradable and can be accumulated in plants and living organisms (Li et al. 2016a). In Europe, the production of the sewage sludge is estimated at 10 million tons of dry weight per year (“Milieu Ltd. Final report for the European Commission” 2008). It has been estimated that the production of SL will increase in the near future due to the industrial and urbanization development. The principal method of SL management is landfilling. Unfortunately, in this way, valuable components of sewage sludge (e.g., phosphorous, nitrogen, organic matter and micronutrients) (Pathak et al. 2009) are depleted. The application of SL to the soil as a fertilizer or soil conditioner would be a better solution. However, a solution is necessary which would utilize the benefits given by SL, with a simultaneous reduction in the risk associated with the presence of PTEs in it. One of the possible solutions would be to incorporate into the soil, together with SL, a material characterized by high affinity for PTEs. Such a solution would result in selective binding of PTEs and in consequence reduce their migration in the soil and accumulation by plants and soil organisms. Biochars could be such material (Ahmad et al. 2014; Mohan et al. 2014; Rajapaksha et al. 2016; Inyang et al. 2016).

Our previous studies (Oleszczuk et al. 2012, 2014) showed that mixing biochar with sewage sludge reduces the bioavailable fraction of polycyclic aromatic hydrocarbons (PAHs) in the sewage sludge. Furthermore, a lot of studies demonstrated that BCs have a great potential also in the adsorption of PTEs (Beesley and Marmiroli 2011; Deveci and Kar 2013; Tytłak et al. 2015; Bogusz et al. 2015; Usman et al. 2016). High adsorption capacity of BC is connected with the presence of a large amount of oxygen-containing groups (e.g., carboxyl, hydroxyl, phenolic) on the BCs surface as well as a high specific surface area and porosity of BC (Mohan et al. 2007). High sorption capacity of BC to PTEs was used as a low-cost remediation strategy for soils and mine tailings contaminated by PTE (Fellet et al. 2011; Karami et al. 2011; Kelly et al. 2014). For example, Kelly et al. (2014) and Fellet et al. (2011) studied the influence of the application of BC to mine sites on the concentration and bioavailability of PTEs. Authors observed the reduction in the concentration of bioavailable Cd, Pb, Tl and Zn (Fellet et al. 2011) as well as decrease in leached Zn and Cd after biochar application (Kelly et al. 2014). Karami et al. (2011) determined also the influence of compost and BC on mobility of Pb and Cu in soil. BC decreased Cu content in pore water and Cu and Pb content in the shoots of ryegrass (*Lolium perene*) (Karami et al. 2011). Hence, the application of the BC with the SL to the soil may also be a good solution for immobilization of PTEs in the SL-amended soil. The determination of mechanisms responsible for adsorption of PTE ions by soil, sewage sludge, biochar and a mixture of these materials forms the basis for estimating factors that affect the bioavailability of metals in sewage sludge- and biochar-amended soil.

The aim of the presented study was the evaluation of the influence of biochar (BC) addition to the sewage sludge (SL) and subsequently to soil on the sorption capacity of SL or SL/BC-amended soil. The adsorption capacity of four different metal ions [Cd(II), Cu(II), Ni(II), Zn(II)], very common in sewage sludge, was investigated. Furthermore, the desorption of investigated metal ions from studied materials was also investigated to evaluate the strength of bonding between PTE and fertilized soil.

## Materials and methods

### Materials

The soil (S) used in the experiment was collected from Bezek near Chełm city (N: 51°12′02″ E: 23°17′37″). It was a podzolic soil (S), classified as a V class of soil and poor rye soil complex. Sewage sludge (SL) was collected during spring 2014 from municipal sewage treatment plant localized in Chełm (51°07′56″N 23°28′40″E). Sewage treatment plants were located on the agricultural areas and used mainly municipal wastewater without the significant influence of the industry. BC was a commercial available biochar and was produced from willow at 600 °C in limited oxygen conditions (1–2%) and provided by Fluid SA (Sędziszów, Poland). Physicochemical properties of control soil, sewage sludge, biochar and their mixtures are presented in Tables 1 and 2.

**Table 1:** Physicochemical properties of the control soil (S) and mixed materials

	pH	H_h_	CEC	TOC	DOC	N	Ash	*S*_BET_	*V*_p_ (·10^−3^)	PS	*S*_micro_	*V*_micro_ (·10^−4^)
S	5.3	21.8	3.78	0.61	17.0	0.072	98.25	1.054	3.9	14.94	0.745	3.8
S + SL	4.6	29.0	3.25	0.71	16.9	0.082	97.88	0.859	3.6	16.56	0.331	1.6
S + SL + BC2.5	4.4	35.6	3.25	0.66	18.7	0.082	96.92	0.548	3.6	26.32	0.722	3.7
S + SL + BC5.0	4.5	31.9	3.20	0.69	12.8	0.079	97.77	0.619	2.7	17.50	0.783	3.2
S + SL + BC10	4.7	29.0	3.73	0.67	14.2	0.082	97.75	0.777	3.5	18.06	0.312	1.3

pH in KCl, *H*_h_ hydrolytic acidity (mmol(+)/kg of dry–wet), *CEC* cation exchange capacity (meq/100 g), *TOC* total organic carbon content (%), *DOC* dissolved organic carbon (mg/L), *N* the contribution (%) of nitrogen, *Ash* ash content (%), *S*_BET_ specific surface area (m^2^/g), *V*_p_ pore volume (cm^3^/g), *PS* pore size (nm), *S*_micro_ micropore area (m^2^/g), *V*_micro_ micropore volume (cm^3^/g)

**Table 2:** Physicochemical properties of sewage sludge (SL) and biochar (BC)

	pH	Elemental composition				TOC	Ash	H/C	O/C	(O + N)/C	C/N	*S*_BET_	*V*_p_	PS	*S*_micro_	*V*_micro_ (·10^−4^)
		C	H	N	O											
SL	6.74	35.18	5.48	4.60	23.96	29.6	30.78	0.16	0.68	0.81	7.65	0.290	0.0025	34.60	0.189	0.7
BC	8.90	52.2	2.23	1.13	19.32	31.8	25.12	0.043	0.37	0.39	46.20	5.262	0.010	7.59	4.112	16.8

pH in KCl, *CHNO* the contribution (%) of carbon, hydrogen, nitrogen and oxygen, *TOC* total organic carbon content (%), *Ash* ash content (%), *H/C* ratio of hydrogen to carbon, *O/C* ratio of oxygen to carbon, *(O* *+* *N)/C* polarity index, *N/C* ratio of nitrogen to carbon, *S*_BET_ specific surface area (m^2^/g), *V*_p_ pore volume (cm^3^/g), *PS* pore size (nm), *S*_micro_ micropore area (m^2^/g), *V*_micro_ micropore volume (cm^3^/g)

Following experimental variants were prepared: (1) control soil without amendments (S); (2) sewage sludge (10 t_dw_/ha) and soil (S + SL); (3) sewage sludge mixed with 2.5% of biochar and with soil (S + SL + BC2.5), (4) sewage sludge mixed with 5% of biochar and with soil (S + SL + BC5.0) and (5) sewage sludge mixed with 10% of biochar and with soil (S + SL + BC10). Methods of materials characterization and description of material properties are presented in Supporting Information (SI).

### Batch sorption experiment

The adsorption process of Cd(II), Cu(II), Ni(II) and Zn(II) was conducted under pH of 5.5, to eliminate the possibility of the precipitation of metal hydroxides in the volumetric phase. The PTEs hydroxides precipitate under following pHs 8, 6, 7 and 6.5, for Cd(OH)_2_, Cu(OH)_2_, Ni(OH)_2_ and Zn(OH)_2_ (Charlot and Bezier 1957).

The effect of time on adsorption of PTEs onto adsorbent materials was carried out versus time intervals up to 24 h. The initial concentration of Cd(II), Cu(II), Ni(II) and Zn(II) ions in the single element solutions was 100 mg/L (each component separately). The optimal pH was adjusted to be 5.5 for all metal ions. Kinetics solutions were agitated on a shaker (358A, Elpin +, Poland) at 120 rpm constant speed at room temperature 22 ± 2 °C. Next, mixtures were separated by centrifugation (MPW-56, MPW Med. Instruments, Poland), and the filtrates were analyzed for the PTEs concentration.

The adsorption isotherms of Cd(II), Cu(II), Ni(II) and Zn(II) were performed in the single component systems at initial pH of 5.5. The 0.2 g ± 0.03 g of each sorbent was mixed with 50 mL solutions of either Cd(II), Cu(II), Ni(II) or Zn(II) in the range of concentrations from 5 to 600 mg/L. The solutions were agitated on a shaker (120 rpm) (358A, Elpin + , Poland) with temperature of 22 ± 2 °C for a specific period of contact time determined on the basis of kinetics study. After shaking, the samples were filtered and the concentration of Cd(II), Cu(II), Ni(II) and Zn(II) was measured in the supernatant.

Desorption study with distilled water versus time interval was also investigated. All studied materials with known amount of loaded Cd(II), Cu(II), Ni(II) or Zn(II) ions were applied. To obtain the materials with loaded heavy metal ions, the 0.3 g ± 0.05 g of each sorbent was mixed with 50 mL solutions of either Cd(II), Cu(II), Ni(II) or Zn(II) at the concentration of 150 mg/L. The solutions were mixed for 24 h. After 24 h, solid phase was separated from solution by filtration and the concentrations of PTEs in solution were measured and the adsorbed concentration of PTEs was calculated. Solid phase was dried before using. Materials loaded with Cd(II), Cu(II), Ni(II) or Zn(II) about 0.2 g ± 0.03 g were placed into Erlenmeyer flask and mixed with 50 mL of distilled water. Suspensions were agitated in shaker (120 rpm) (358A, Elpin +, Poland) at room temperature of 22 ± 2 °C for 160 h.

Final suspensions after particular experiments (kinetics, isotherms, desorption) were centrifuged, filtered, and the supernatant solution was analyzed regarding to Cd(II), Cu(II), Ni(II) and Zn(II) ions using the flame atomic absorption spectrometer Varian Spectra AA-880 (Carl Zeiss, Jena, Germany) with hollow cathode lamp HCL (Varian). Detailed information about metal ions determination and kinetics and isotherm data calculations is presented in SI.

## Results and discussion

### Adsorption of PTE by soil, sewage sludge and biochar

**Fig. 1:**
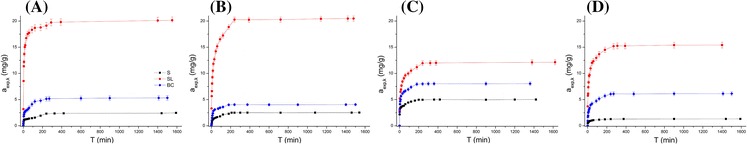
Adsorption kinetics of Cd(II) (**A**), Cu(II) (**B**), Ni(II) (**C**), Zn(II) (**D**) ions onto S, SL and BC; *m* = 0.2 g ± 0.03 g, *V* = 50 mL, C_ions_ = 100 mg/L, pH: 5.5, *T* = 22 ± 2 °C

Figure 1 shows the kinetics of adsorption of the studied metals by the soil, SL and BC. SL required the longest time to reach adsorption equilibrium (3.5–5 h), followed by BC (3–3.5 h), while this time was shortest for the control soil (1.5–3 h) (Table 3). This was probably due to the composition of the materials tested, which is the most heterogeneous in the case of SL, followed by BC, whereas it was the least heterogeneous for the soil.

**Table 3:** Parameters of PTE sorption isotherms fitted with Langmuir and Freundlich models, and sorption kinetics fitted to PSO

Metal	Sample	*a*_exp. *i*_	Isotherm models						*a*_exp, *k*_	Kinetic model		
			Langmuir			Freundlich				PSO		
			*K*_L_ (·10^−3^)	*a*_m_	*R* ^2^	*K* (·10^−2^)	*n*	*R* ^2^		*k*_2_	*R* ^2^	*a*_eq_
Cd	S	6.79	3.6	11.1	0.564	1.46	0.64	0.948	2.46	0.56	0.998	2.47
	S + SL	8.32	4.0	13.5	0.522	3.31	0.52	0.915	3.27	0.72	1.000	3.31
	S + SL + BC2.5	9.42	59.0	13.3	0.961	4.19	0.34	0.990	4.76	3.11	1.000	4.77
	S + SL + BC5.0	8.16	12.6	9.3	0.921	3.92	0.54	0.938	4.28	15.10	1.000	4.30
	S + SL + BC10	7.21	11.4	8.6	0.948	3.79	0.70	0.966	4.01	20.50	1.000	4.01
	SL	42.26	573.0	48.1	0.988	5.53	0.31	0.928	20.2	445.00	1.000	20.20
	BC	10.89	90.0	11.1	0.995	15.30	0.37	0.694	5.33	8.38	1.000	5.39
Cu	S	3.33	10.3	3.5	0.977	4.47	0.48	0.991	2.52	0.96	1.000	2.55
	S + SL	4.62	28.6	5.5	0.914	5.10	0.39	0.971	2.61	1.78	0.999	2.65
	S + SL + BC2.5	5.22	9.0	6.7	0.771	4.72	0.46	0.793	2.34	4.18	1.000	2.36
	S + SL + BC5.0	5.01	8.5	5.9	0.831	3.53	0.52	0.939	2.44	17.90	1.000	2.43
	S + SL + BC10	4.34	6.1	5.1	0.854	2.80	0.53	0.984	2.08	21.30	1.000	2.08
	SL	27.02	39.3	28.9	0.990	18.20	0.34	0.962	20.5	615.00	1.000	20.70
	BC	11.37	20.1	11.7	0.997	15.30	0.38	0.554	4.05	15.40	1.000	4.09
Ni	S	6.88	4.8	8.0	0.984	1.13	0.70	0.986	5.02	18.70	1.000	5.04
	S + SL	7.79	5.3	11.4	0.916	1.46	0.52	0.986	5.34	16.20	1.000	5.89
	S + SL + BC2.5	8.01	4.9	13.8	0.778	1.00	0.70	0.978	5.08	23.60	1.000	5.11
	S + SL + BC5.0	6.73	4.37	10.21	0.899	0.99	0.73	0.988	3.54	32.50	1.000	3.59
	S + SL + BC10	5.75	3.47	8.50	0.911	0.62	0.78	0.984	4.02	42.00	1.000	4.03
	SL	19.12	26.51	20.37	0.996	5.57	0.49	0.944	12.1	759.00	1.000	12.20
	BC	9.82	18.09	11.11	0.975	5.51	0.50	0.972	8.05	76.90	1.000	8.09
Zn	S	3.20	6.13	3.69	0.884	2.99	0.49	0.918	1.32	0.25	1.000	1.32
	S + SL	4.00	6.39	4.73	0.915	3.62	0.32	0.982	1.86	0.34	0.999	1.89
	S + SL + BC2.5	5.02	8.97	6.09	0.883	2.75	0.52	0.989	2.43	2.31	1.000	2.43
	S + SL + BC5.0	4.66	8.71	4.99	0.950	2.69	0.57	0.980	3.02	3.53	1.000	3.03
	S + SL + BC10	4.23	7.68	4.68	0.979	2.45	0.65	0.990	2.83	2.76	1.000	2.85
	SL	29.54	37.1	30.58	0.989	6.71	0.41	0.897	15.4	371.00	1.000	15.60
	BC	9.62	10.26	17.48	0.983	7.60	0.45	0.892	6.14	7.22	1.000	6.20

*a*_m_ the maximal theoretically adsorbed amount (sorption capacity) (mg/g), *K*_L_ the Langmuir constant, *a*_exp.*i*_ the maximal experimental amount adsorbed at equilibrium time (mg/g), *K*, *n* empirical constants indicative of sorption capacity and sorption intensity, *a*_exp,*k*_ maximal adsorption capacity achieved in the kinetic study (mg/g), *a*_eq_ the amount adsorbed at equilibrium time (mg/g), *k*_2_ (g/mg min) the rate constants of pseudo-second-order equation, *R* regression coefficient

Among three models tested (PFO, PSO, Elovich), the best fit of the experimental data was obtained for the PSO model (Table 3). Based on the k_2_ values of the PSO model, it can be concluded that the adsorption equilibrium rate for the individual metals, regardless of the type of matrix (soil, SL, BC), was as follows: Zn < Cd < Cu < Ni. Such an order can be explained based on the hydrated radius of the metal ions (Table S5). When the radius increases, the adsorption decreases (Guzel et al. 2008). The affinity of these ions for oxygen- and nitrogen-containing groups also contributes to faster kinetics in the case of Ni(II) and Cu(II) (Uchimiya et al. 2010). Other authors’ studies on sorption of PTEs by sewage sludge (Phuengprasop et al. 2011; Agrafioti et al. 2014), biochar (Inyang et al. 2012; Kolodynska et al. 2012) and soils (Aşçı 2012) also confirm that the best fit of experimental data is obtained for the PSO model. On the other hand, comparing the time necessary to reach equilibrium by the particular metals, it varied depending on the metal in question or the type of material applied. Generally, it was within the range observed by other authors (Agrafioti et al. 2013, 2014; Aşçı 2012; Chen et al. 2011; Devi and Saroha 2017).

**Fig. 2:**
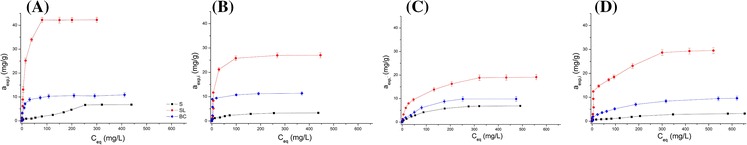
Initial runs of adsorption isotherms of Cd(II) (**A**), Cu(II) (**B**), Ni(II) (**C**), Zn(II) (**D**) ions onto S, SL and BC; *m* = 0.2 g ± 0.03 g, *V* = 50 mL, pH: 5.5, *T* = 22 ± 2 °C, *t* = 24 h

Figure 2 shows the isotherms of adsorption of the studied PTEs by the soil, SL and BC. Their adsorption capacity varied and was directly related to the physicochemical properties of these materials. SL exhibited the highest sorption capacity toward all PTEs studied, followed by BC, while the lowest sorption was found for the soil.

Depending on the material investigated, the fit of the experimental data to the models tested varied (LM, FM, TM). For the soil, the best fit of the experimental data was observed for FM (*R*^2^ > 0.918). In the case of SL and BC, on the other hand, LM best reflected the experimental data (*R*^2^ > 0.975). This observation was confirmed for all PTEs studied. The best fit to the FM model demonstrates that in the soil the process of adsorption occurred on a heterogeneous surface, which is also indicated by the irregular shape of the adsorption isotherms (Covelo et al. 2007). The energy heterogeneity of soil surface had been previously confirmed by Asci et al. (2008). In turn, the fit of the experimental data to the LM model for SL and BC reveals that the process of adsorption of the studied PTEs by these materials occurs predominantly through chemisorption (Kizito et al. 2015). Similar values of the experimentally obtained maximum adsorption capacity, a_exp,i_ and a_m_, derived from LM both for SL and BC (Table 3), are evidenced that the monolayer was almost completely filled with the adsorbed metal ions. Furthermore, it is worth noting that in comparison with the other PTEs, the high value of the *K*_L_ coefficient for Cd sorption on sewage sludge, which was 573 L/mg (Table 3), shows high affinity of this metal for SL surface and indicates strong binding of this metal to SL surface, which will explain the weakest desorption of Cd from SL compared to other PTEs in a subsequent study.

The higher adsorption capacity of SL and BC compared to the soil is understandable and has been described in a number of publications, while the differences between BC and SL need to be explained in more detail.

The differences in adsorption capacity of SL and BC are undoubtedly determined by the physicochemical properties of these materials (Table 1). Compared to BC, SL was characterized by the presence of a larger amount of oxygen groups, as indicated by the higher molar ratios of O/C and (O + N)/C for SL (Table 2). The presence of oxygen groups plays an important role in adsorption of PTE ions, since these groups participate in complexation of these ions (Li et al. 2017). Harvey et al. (2011) reported that sorption of Cd ions is primarily based on ion exchange and complexation with surface groups (Harvey et al. 2011). Hence, a larger amount of oxygen groups on the surface of SL will determine its higher sorption capacity toward the PTEs studied. Moreover, SL contained more organic matter, on which PTEs are adsorbed more readily, than BC (Antoniadis et al. 2007). Another factor contributing to the differences in adsorption of PTEs by SL and BC is the presence of sulfur, phosphorus and nitrogen in them. Higher contents of these elements were determined in the SL structure compared to BC, which was translated into a higher adsorption capacity of SL toward the PTEs studied, since PTEs are characterized by high constants of complexation (pK) (Table S5) with phosphorus- and sulfur-containing groups (Charlot and Bezier 1957). Moreover, a higher content of silicon in organic form (siloxane, silicone) in the SL structure compared to BC (Table S3) could also have been a factor that increased the adsorption capacity of SL relative to BC. A previous study (Liu et al. 2000) revealed that organic silica exhibits a significant adsorption capacity toward ions of PTEs.

Identification, based on the XPS spectra, of the presence of absorbed water and oxygen on the BC surface (Table S3), with these molecules blocking a part of adsorption sites and thus reducing the level of adsorption of the metals by BC, is also important information. Furthermore, considering the surface properties, it can be noted that the smaller pore size (PS) of BC made it impossible to capture large molecules of the adsorbates due to their charge and polarity (Ahmedna et al. 2004).

Based on research conducted thus far (Arias et al. 2002; Aşçı 2012; Covelo et al. 2007; Li et al. 2016b), it can be concluded that for soils experimental data are usually best fitted to FM, as observed in the present study. For example, studying the sorption of Cd, Cu, Ni, Pb, Zn by six soils Covelo et al. (2007) found that the data were closely correlated mostly with FM, with few cases of the correlation to LM (Covelo et al. 2007). FM was reported as more suitable for adsorption of PTEs by soils also by other authors (Arias et al. 2002; Aşçı 2012; Li et al. 2016b).

For sewage sludge, Merrikhpour and Jalali (2012) in the case of adsorption of Cd(II), Cu(II), Pb(II) and Zn(II) on industrial sludge and Xie et al. (2013) in the case of adsorption of Cu(II), Pb(II) and Cd(II) on municipal sewage sludge observed better correlation to LM than other models (Merrikhpour and Jalali 2012; Xie et al. 2013). Similarly, for biochars, Kolodynska et al. (2012) noticed that the isotherms data fitted closely to LM in the most cases for six biochars obtained from the pig and cow manure (Kolodynska et al. 2012). The best correlation the isotherm data to LM for biochars was also observed in other reports (Arivoli et al. 2008; Chen et al. 2011; El-Shafey et al. 2002; Park et al. 2013; Usman et al. 2016).

### Adsorption kinetics of sewage sludge-soil or biochar-sewage sludge-soil mixture

**Fig. 3:**
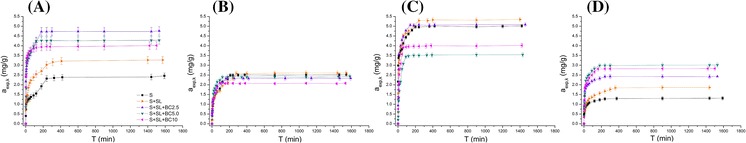
Adsorption kinetics of Cd(II) (**A**), Cu(II) (**B**), Ni(II) (**C**), Zn(II) (**D**) ions onto mixed of S, SL and BC; *m* = 0.2 g ± 0.03 g, *V* = 50 mL, C_ions_ = 100 mg/L, pH: 5.5, *T* = 22 ± 2 °C

Application of SL and SL/BC to the soil affected the change in its adsorption capacity toward all PTEs studied. Adding SL alone to the soil influenced increasing the soil’s adsorption capacity and extending the time required to reach adsorption equilibrium (Fig. 3). The addition of BC to SL and an increase in BC rate in SL resulted in a decrease in the time necessary to achieve adsorption equilibrium compared to both the soil and the SL-amended soil (Fig. 3). It is understandable because BC reached adsorption equilibrium faster than SL. Therefore, adding BC to SL accelerated the kinetics of adsorption of the PTEs, which is extremely important information from the practical point of view.

Similarly as for the soil, SL and BC, in the case of their mixtures the best fitting of the experimental data was also obtained for the PSO model (*R*^2^ > 0.998). The adsorption equilibrium rate (based on *k*_2_, Table 3) for the individual metals did not change (Zn < Cd < Cu < Ni) either. The obtained results are in agreement with a previous study conducted by Paranavithana et al. (2016), who observed that for Cd and Pb adsorption on soil amended with coconut shell-derived biochar; the best fit of experimental data is obtained for the PSO model (Paranavithana et al. 2016). Up to now, this has been the only study to investigate the kinetics of adsorption by biochar-amended soil.

### Adsorption isotherms of sewage sludge-soil or biochar-sewage sludge-soil mixture

**Fig. 4:**
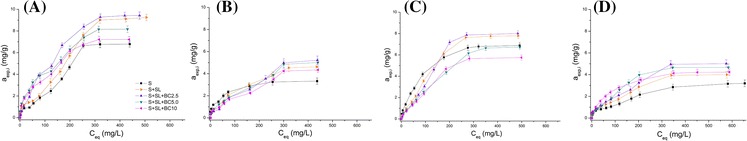
Initial runs of adsorption isotherms of Cd(II) (**A**), Cu(II) (**B**), Ni(II) (**C**), Zn(II) (**D**) ions onto mixed of S, SL, BC; *m* = 0.2 g ± 0.03 g, *V* = 50 mL, pH: 5.5, *T* = 22 ± 2 °C, *t* = 24 h

Figure 4 shows the isotherms of adsorption of the PTEs studied on the SL-amended soil and on the soil amended with SL with different percentages (2.5, 5 and 10%) of BC, whereas Table 3 shows the adsorption parameters calculated for the individual models. The FM model was characterized by a slightly better fit to the experimental data (*R*^2^ > 0.915) than the LM model (Table 3). This is understandable because the dominant fraction in this treatment is still the soil, in the case of which the best fit to the experimental data was also observed for the FM model. After mixing the materials, physisorption was still the dominant process on the heterogeneous surface due to the dominant proportion of the soil in the mixture, while the chemisorption processes are probably of lesser importance in this case. The heterogeneity of the surfaces in the mixed treatments is also confirmed by the irregular shape of the adsorption isotherms (Fig. 4) (Covelo et al. 2007).

To date, presented research has been the only study which determined the effect of BC addition to SL on adsorption of PTEs by amended soil. Existing research primarily focuses on investigating the adsorption of metals by soils amended with sewage sludge alone (Antoniadis et al. 2007; Hooda and Alloway 1994) or biochar alone (Beesley and Marmiroli 2011; Melo et al. 2016; Uchimiya et al. 2012; Xu and Zhao 2013). Similarly as in the present study, adding soil amendments resulted in the soil’s increased adsorption capacity. The obtained data also best correlated with FM. For example, Antoniadis et al. (2007) studied Cd, Ni and Zn sorption on soil amended with sewage sludge alone and obtained a good fit to FM for all metals studied, both in single treatments and for competitive adsorption (Antoniadis et al. 2007). Hooda and Alloway (1994) studied Cd and Pb adsorption on sewage sludge-amended soil and observed that addition of sewage sludge to the soil increased its sorption capacity. This phenomenon was explained by an increase in organic matter content after application of sewage sludge to the soil. Based on the fit of the adsorption isotherms, it was found that FM best described Cd and Pb sorption both on the control soil and on the sewage sludge-amended soil (Hooda and Alloway 1994). Xu and Zhao (2013) studied Cu, Pb and Cd adsorption on three different soils with the addition (3 or 5%) of two biochars derived from canola straw and peanola straw. Biochar addition contributed to an increase in sorption capacity of the studied soils in proportion to the amendment applied. The experimental data of the adsorption isotherms better correlated with LM (Xu and Zhao 2013). Melo et al. (2016) studied the effect of the addition of biochar (10% w/w) derived from sugarcane straw on the adsorption capacity of two types of soil toward Cd and Zn. They noted that biochar addition had an effect on increasing the soil adsorption capacity and that the experimental data correlated both with FM and LM (Melo et al. 2016).

When comparing the parameters *K* and *n* derived from the FM model (Table 3), it can be stated that they are characterized by certain repeatability for all PTEs studied. Adding SL increased the value of the *K* coefficient for all metals. The greatest effect was found for Cd, followed by Cu, Zn and Ni. The addition of BC to SL, depending on the rate, had a different effect on the value of *K*, depending on the metal. It was, however, observed for all PTEs that an increase in the proportion of BC in SL reduced the value of this parameter. This is in agreement with the observations concerning the sorption of PTEs by the “pure” materials tested, in the case of which BC was characterized by lower affinity for PTEs than SL. Thus, the “dilution” of SL by BC reduced the sorption capacity of SL. It is, however, puzzling that in spite of the lower affinity of BC than SL, the highest affinity for the PTEs studied was found in the treatment with the 2.5% biochar rate (higher even in the case of the soil amended with SL alone). This may be attributable to the complexity of the mechanism of adsorption of the PTEs studied on the mixed materials involving competition between physisorption and chemisorption, as indicated by the high regression coefficients both for LM and FM for the mixed materials (Table 3). The highest adsorption capacity of S + SL + BC2.5 can be also explained by the presence of the dissolved organic carbon (DOC) (Table 1). It was reported (Christensen et al. 1996) that DOC is able to complex the PTEs ions such as Cd, Ni, Zn. Bolton and Evans (1991) also noted that DOC in poultry litter encouraged the formation of complexes with organic ligands and trace elements presented in the soil. Moreover, it was reported that organic matter exhibited a high affinity for Cu (Shaheen et al. 2013). On the basis of this information, it can be stated that the highest value of DOC in the case of S + SL + BC2.5 was also responsible for the highest adsorption capacity of this material in respect of studied metal ions. Moreover, the highest PS, *V*_p_ and *V*_micro_ of S + SL + BC2.5 in comparison with other SL/BC systems should also be considered (Table 1). High values of the mentioned factors may affect better transfer of PTEs ions into pores.

Adding SL to the soil resulted in a decrease in the *n* value in the case of all PTEs, which evidences the soil’s increased adsorption heterogeneity. In most cases (except for Cd), adding BC to SL caused an increase in the *n* coefficient. Moreover, with increasing contribution of BC in SL, the *n* value increased, indicating reduced adsorption heterogeneity. To determine the physicochemical properties that might potentially affect the sorption of PTEs in the treatment with sewage sludge amendment and sewage sludge with biochar addition, a statistical analysis of *K* and *n* was performed in relation to the physicochemical properties of the mixed materials (Table S8). But no relationships were found that would allow us to explain the specific observations.

### Desorption study

**Fig. 5:**
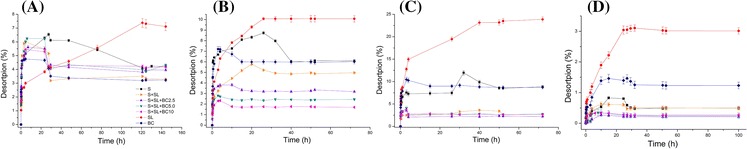
Desorption kinetics of Cd(II) (**A**), Cu(II) (**B**), Ni(II) (**C**) and Zn(II) (**D**) from S, SL and BC and mixed of S, SL, BC by distilled water; *m* = 0.2 g, *V* = 50 mL, *A* = 2 ± 0.20 mg/g, *T* = 22 ± 2 °C

Figure 5 shows the kinetics of desorption of the PTEs studied from the soil, SL, BC, SL-amended soil and SL/BC-amended soil. Except for SL, desorption of the investigated ions was two level. During the first phase, the metal ions were desorbed to reach a certain maximum and subsequently they were re-adsorbed on the surface, as evidenced by a decrease in desorption after the maximum value was reached. This effect was particularly visible in the treatments with BC (Fig. 5). A similar relationship was not observed only for SL, which is evidenced that the desorbed ions were probably not re-adsorbed on its surface. It can therefore be inferred that despite the desorption of metal ions from the soil, SL-amended soil, SL/BC-amended soil and BC alone, a part of ions is re-adsorbed.

The largest range of desorption, depending on the type of metal from 3 to 25%, was observed for SL. This indicates the weakest binding of the metals by this material. Although SL was characterized by the best adsorption capacity, in its case the strength of these bonds is weakest. This shows that BC addition to SL may be an important factor that reduces the mobility and bioavailability of PTEs in sewage sludge-amended soils.

Ni was bound weakest by SL and the other materials tested, whereas Zn was bound strongest (Fig. 5). This was associated with the fact that among the PTEs studied, Ni was characterized by the lowest hydrated radius (Table S5) and hence the strength of its interactions with the surface of the sorbents was lowest. Zn, in turn, was characterized by the highest value of the hydrated radius among the metals studied (Table S5), and therefore it is more difficult to remove its ions from the surface of the adsorbents. The dominant mechanism of adsorption of Ni(II) and Zn(II) ions is also an important issue. In the case of Ni, it is primarily complexation with surface ions (Uchimiya et al. 2011). Bonds of this type are not stable and they can be broken more easily compared to surface precipitation, which was the dominant process for Zn(II) binding. Moreover, statistically significant correlations were found between the physicochemical properties of the materials tested and maximum desorption of Ni(II) and Zn(II) (Table S9). For both metals, a positive relationship was observed between pH and desorption. This may be related to the fact that with increasing pH the surface charge of the materials changes, which allows greater desorption of the metal ions. Furthermore, a negative relationship of TOC and N with desorption of Ni(II) ions was revealed. This indicates the preferred mechanism of adsorption of Ni(II) ions, i.e., complexation with both nitrogen-containing groups and organic groups (pyrone, pirydine, etc.), as mentioned before. The fewer of these groups (a lower value of TOC and N), the weaker interactions, can be broken more easily, on which the adsorption process is based. A positive relationship was found between *S*_BET_ and Zn(II) desorption (Table S8). This may be related to the fact that with increasing specific surface area the hydrated Zn(II) ions were less tightly packed, and thereby access of the desorbing reagent was better, which was associated with increased desorption.

Moreover, the control soil (*S*) and BC were also characterized by high desorption of ions. Nevertheless, in all cases studied, mixing SL with the soil resulted in decreased desorption of these ions compared to the materials considered separately. Adding SL/BC to the soil had an even greater impact on reducing desorption of the metal ions studied. Cd was an exception because in its case it was difficult to observe any trend. It should also be stressed that only in the case of Cu a relationship was observed where an increase in biochar rate was accompanied by decreased desorption of this metal. In the other cases, biochar rate did not generally affect the desorption range. However, almost every time (except for Cd) much lower desorption was found for the treatments with biochar than for the sewage sludge-amended soil.

## Conclusions

Increased amounts of sewage sludge produced force to develop new solutions for utilization of this type of materials. Application of sewage sludge to soil, on the one hand, improves physicochemical properties of soils and enhances crop productivity, but on the other hand, contaminants such as PTEs are introduced into the soil together with sewage sludge. A solution is to incorporate biochar into the soil together with sewage sludge, as biochar will cause immobilization of PTEs and reduce their mobility. Based on the present study, it can be concluded unambiguously that the 2.5% addition of biochar to sewage sludge increased the soil’s sorption capacity toward the PTEs studied. After application of the biochar, desorption of PTEs from the sewage sludge and from the control soil decreased, which also suggests that the mobility of PTEs was reduced. Thus, the incorporation of sewage sludge with biochar addition into the soil reduces the risk of contamination of the environment with PTEs and increases the safety of soil amendment with sewage sludge. Therefore, biochar incorporated with sewage sludge decreases potential desorption of the metals studied, which significantly reduces environmental risks related to their presence in the environment. This is an important issue because the reduction in PTEs desorption from sewage sludge reduces the amount of toxic elements entered to the food chain. Immobilization of PTEs after the application of biochar prevents migration of heavy metals to plants, water and next to living organisms. As can be seen, the binding of metals by sewage sludge is unstable, which may in the future result in desorption of metals into the environment. Thus, the use of biochar as an addition to sewage sludge can be a new and interesting solution in future utilization of sewage sludge.

## Electronic supplementary material

Below is the link to the electronic supplementary material.

Supplementary material 1 (DOCX 199 kb)
