# Supplementary material for: Adsorption and desorption of heavy metals by the sewage sludge and biochar-amended soil
Source: Environ Geochem Health. 2017 Nov 7;41(4):1663–74. doi: 10.1007/s10653-017-0036-1 (PMC6751146; doi:10.1007/s10653-017-0036-1)
Supplement: Supplementary file 1 — Supplementary material 1 (DOCX 199 kb) [file 10653_2017_36_MOESM1_ESM.docx]

**Supplementary material**

**ADSORPTION AND DESORPTION OF HEAVY METALS BY THE SEWAGE SLUDGE AND BIOCHAR-AMENDED SOIL**

Aleksandra Bogusz^1^, Patryk Oleszczuk^1*^, Ryszard Dobrowolski^2^

^1^Department of Environmental Chemistry, Faculty of Chemistry, Maria Sklodowska-Curie University, Maria Curie-Sklodowska Square 3, 20-031 Lublin, Poland

^2^Department of Analytical Chemistry, Faculty of Chemistry, Maria Skłodowska-Curie University, Maria Curie-Skłodowska Square 3, 20-031, Lublin, Poland

*Correspondence to: Patryk Oleszczuk, Department of Environmental Chemistry, University of Maria Skłodowska-Curie, pl. M. Curie-Skłodowskiej 3, 20-031 Lublin, Poland, tel. +48 81 5375515, fax +48 81 5375565; e-mail: [patryk.oleszczuk@poczta.umcs.lublin.pl](mailto:patryk.oleszczuk@poczta.umcs.lublin.pl)

**^*^Corresponding author:** e-mail: patryk.oleszczuk@umcs.lublin.pl

The following are included as supplementary information for current paper:

Number of tables: 9

Number of figures: 1

**MATERIALS AND METHODS**

**Chemicals**

The initial standard stock solutions of Cd(II), Cu(II), Ni(II) and Zn(II) ions (each of 1000 mg/L) were prepared by dissolution of respectively Cd(NO_3_)_2_·4H_2_O, Cu(NO_3_)_2_·3H_2_O, Ni(NO_3_)_2_·6H2O and Zn(NO_3_)_2_·6H_2_O powder (POCH, Gliwice, Poland) in a redistilled water. The calibration curves of determined ions were established using the standard solutions of Cd(II), Cu(II), Ni(II) and Zn(II) prepared in 0.5 mol/L HNO_3_ by dilution from stock solution each of 1000 mg/L (Merck, Darmstadt, Germany).

**Characterization of soil, sewage sludge and biochar**

The pH of each material was measured potentiometrically using 1 mol/L potassium chloride after 24 hours in the liquid/solid ratio of 10. The TOC-VCSH (SHIMADZU) with Solid Sample Moduls (SSM-5000) was applied for the total organic carbon content (TOC) determination. The total nitrogen (N_t_) was measured by using the Kjeldahl’s procedure without the application of Dewarda’s alloy (Cu-Al-Zn alloy- reducer of nitrates and nitrites). FT-IR/ATR spectrum of the materials sample was recorded by means of the FTIR Nicolet 8700A spectrometer equipped with Smart Orbit TR diamond ATR (in the helium atmosphere in a detector) at RT over the 4000–400 cm^−1^ range at the resolution of 4 cm^−1^ and maximum source aperture. The spectrum was normalized by computing the ratio of a sample spectrum to the spectrum of a MTEC carbon black standard. Interferograms of 1024 scans were averaged for the spectrum. The low-temperature (77.4 K) nitrogen adsorption-desorption isotherms were used for identify the structure of studied materials. The data were obtained with Micromeritics ASAP 2405 N adsorption analyzer. In the base of the standard BET method the specific surface areas S_BET_ were calculated. The carbon, hydrogen and nitrogen content was determined with the CHN Elemental Analyzer (Carlo-Erba NA-1500) via high-temperature catalyzed combustion follow by infrared detection of resulting CO_2_, H_2_ and NO_2_. The X-ray photoelectron spectroscopy were used for surface elemental composition characterization.

**Detection of PTEs**

Concentrations of the PTEs in adsorption system were analyzed using the flame atomic absorption spectrometer VARIAN Spectra AA-880 (Carl Zeiss, Jena, Germany). For the measurement of Cd(II), Cu(II), Ni(II) and Zn(II) ions equilibrium concentration the hollow cathode lamp (Varian) was used. The basic parameters of PTEs ions determination are assembled in **Table S1**. The adsorption value in the equilibrium state on studied sorbents was calculated using the following equation:

$a_{exp}=\frac{\left( c_{i}-c \right)\cdot V}{m}$ (1)

where *c_i_* is the initial concentration of PTEs ions in the solution [mg/L], *c* is the equilibrium concentration of PTEs ions in the solution [mg/L], *V* is the volume of the initial solution [L] and *m* is the mass of the biochar [g].

**Data analysis**

The kinetics data were analyzed using Lagergren pseudo – first – order equation (PFO), pseudo – second – order equation (PSO) and Elovich model (EM). For the modeling of the rate the following equations were used:

 (2)

 (3)

where *a_eq_* is the amount adsorbed [mg/g] at equilibrium time [mg/g], *a_t_* is the amount adsorbed [mg/g] at any time t, *t* is contact time [min], *k_1_* [1/min] and *k_2_* [g/mg·min] are the rate constants of the pseudo – first – order equation and pseudo – second – order equation, respectively.

Elovich model (EM) described the chemical adsorption processes on the heterogeneous surfaces. EM is given by the following equation:

$a_{t}=\frac{1}{\beta}ln\left( \alpha\beta\right)+\frac{1}{\beta}ln\left( t \right)$ (4)

where *a_t_* is adsorption at time *t* [mg/g], *β* [g/mg] and *α* [mg/g·min] are Elovich constants. *α* gives an information about initial sorption rate, while *β* about desorption (Aharoni and Tompkins, 1970).

Three widely applied models, Freundlich, Langmuir and Temkin models, were used to fit the sorption isotherms in OriginPro 8. LM is used for a quantitative description of adsorbate monolayers formed on the outer surface of the adsorbent. In addition, the Langmuir model describes the equilibrium distribution of metal ions between two phases, liquid and solid. Moreover, the following model established the existence of homoenergetic adsorption sites on the surface of the adsorbent and the lack of movement of the adsorbate in the plane (Langmuir, 1918) (Samadi et al., 2015). The linear form of equation for Langmuir model can be written:

$\frac{C_{eq}}{a}=\frac{1}{K_{L}a_{m}}+\frac{C_{eq}}{a_{m}}$ (5)

where *a* is the PTEs ions amount adsorbed onto biochars surface [mg/g] at the equilibrium concentration of examined ions *C_eq_* [mg/L], *a_m_* is known as maximum adsorbed amount required to form a monolayer on the material surface (sorption capacity), *K_L_* is the Langmuir constant related to sorption energy. The *K_L_* and *C_eq_* values characterized the Langmuir adsorption model.

The Freundlich adsorption model, in contrast to the LM, describes a process of adsorption extending on heterogeneous surface adsorobentu. Theory of Freundlich adsorption says that the ratio of the amount of solute adsorbed onto the adsorbent mass to the concentration of the solute is not constant in the solution at various concentrations. The heat of adsorption decreases with increasing amounts adsorbed substance (Agrawal et al., 2004). The linear form of Freundlich model is given by following equation:

$\ln a=\ln\left(a_{m}K\right)+n\ln C_{eq}$ (6)

where the Freundlich constant *n* (0≤n≤1) indicates the intensity of adsorption and characterizes the quasi Gaussian energetic heterogeneity of the adsorption system.

Temkin adsorption model has in its description factor includes the interaction between adsorbent and adsorbate. This model established that the heat of adsorption of the molecules in the layer of adsorption decreases linearly with increasing surface coverage (taking into account the interaction of molecules of adsorbent and adsorbate). It is also assumed that the adsorption can be characterized by a uniform distribution of the binding energy, up to a maximum value of this energy (Aharoni and Ungarish, 1977; Samadi et al., 2015). The linear form of Temkin model can be rewritten by following equation:

$a_{eq}=\frac{RT}{b}\ln K_{T}+\frac{RT}{b}\ln C_{eq}$ (7)

where *B= RT/b*, *R* is the gas constant (8.314 J/mol K), *T* is the absolute temperature (K). The constant *b* describes the adsorption heat (kJ/mol), *K_T_* is the constant of equilibrium binding (L/g) related to the maximum binding energy.

**RESULTS AND DISCUSSION**

**Physico – chemical properties of soil, sewage sludge and biochar**

The soil was characterized by a weak acidic pH and was classified as a clay sand in the granulometric composition (Table 1). The cation exchange capacity (CEC) was 3.78 meq/100g and was typical for this kind of soils. The control soil was also characterized by a low content of the total organic carbon (TOC) (0.61%), dissolved organic carbon (DOC) (17.0 mg/L) and total nitrogen (Nt) (0.072%). Molar ratio of TOC/Nt which gives an information about the intensity of the transformation of the organic matter at the value of 8.5 indicates a high degree of humification of the organic matter in the studied soil and a rapidly progressing mineralization process. The control soil was characterized by low surface area (S_BET_) (1.054 m^2^/g), micropore volume (V_micro_) (3.8·10^-4^ cm^3^/g) and micropore area (S_micro_) (0.745 m^2^/g). The pore size at the level of 14.94 nm suggesting that mainly narrow mesopores dominated in the soil. On the basis of XPS data (Table S3) it can be concluded that in the control soil dominated the oxygen (50At%) and silicone (26.4At%). Moreover, the content of carbon (14At%), Al in form of Al_2_O_3_ (5.9At%) and Fe (1At%) was also determined. The contribution of each forms of studied elements were presented in the Table S3. On the basis of Table S4 can be stated that soil contained the highest content of Mn (216.90 mg/kg), Al (904.62 mg/kg) and Fe (3947.43 mg/kg). Lower values (below 20 mg/kg) was determined for Zn (19.42 mg/kg), Cr (13.26 mg/kg), Pb (13.75 mg/kg), Ni (4.12 mg/kg), Cu (2.43 mg/kg), Co (2.34 mg/kg) and the lowest for Cd (0.83 mg/kg).

The sewage sludge (SL) was characterized by pH 6.74. The TOC content was 29.6%. Sewage sludge was characterized by the high content of ash (30.8%). The elemental composition of sewage sludge was as follows C (35.18%)>O (23.96%)>H (5.48%)>N (4.60%). On the basis of Table S4 can be stated that SL contained the highest content of Zn (624.13 mg/kg), Al (854.59 mg/kg) and Fe (4506.76 mg/kg). Lower values (below 100 mg/kg) was determined for Cu (75.80 mg/kg), Mn (74.18 mg/kg), Pb (23.12 mg/kg), Cr (18.67 mg/kg), Ni (12.84 mg/kg), Co (2.49 mg/kg) and the lowest for Cd (1.53 mg/kg).

On the basis of O/C and (O+N)/C ratios (Table 2), sewage sludge can be estimated as hydrophilic and polar material. Considering the structural properties, sewage sludge had a low S_BET_ (0.29 m^2^/g), S_micro_ (0.189 m^2^/g) and V_micro_ (0.7·10^-4^ cm^3^/g). On the basis of the pore size (34.90 nm) it can be stated that, in the structure of the SL the mesopores were dominated (Table 2). The XPS study (Table S3) suggested that in the composition of the SL carbon dominated (82.7At%). The content of the oxygen was 11.5At%, while the nitrogen content was 1.5At%. The contribution of each forms of studied elements were presented in the Table S3. Biochar (BC) was characterized by pH 8.90. The content of TOC was 31.8%. BC was characterized by the high ash content (25.12%). The elemental composition of the BC was as follow C (52.2%)>O (19.32%)>H (2.23%)>N (1.13%). Considering the ratios of O/C and (O+N)/C it can be stated that BC was less hydrophilic and less polar comparing to sewage sludge. Moreover, the low value of H/C suggests that biochar had a high level of carbonization and more aromatic structure than sewage sludge. Furthermore, structural properties indicated that BC had a low specific S_BET_ (5.262 m^2^/g), S_micro_ (4.112 m^2^/g) and V_micro_ (16.8·10^-4^ cm^3^/g). On the basis of S_micro_, V_micro_ and PS (7.59 nm) it can be defined that in the structure of BC the micropores and mesopores dominated. On the basis of XPS study (Table S3) it can be stated that in the elemental composition of BC the carbon dominated (80.5At%). The content of the oxygen was determined as 14At%, while the nitrogen content was 1.1At%. In the comparison to SL, in the composition of BC the forms of sulphur were indetified 0.4At%. The contribution of each forms of studied elements were presented in the Table S3.

**FTIR study of soil, sewage sludge and biochar**

The FTIR spectra of the input materials, S, SL and BC, are shown in Fig S1. These spectra are similar in the wavenumber range of 4000-2000 cm^-1^ and the peaks occur at a similar wave length, but are characterized by different intensity. A broad intense band at a wavenumber of ~3414cm^-1^, the most intense for BC, evidences the presence of –OH groups in the case of all three materials (stretching –OH) and the existence of hydrogen bonds (Yuan et al., 2011). The next peaks occurring at 2917cm^-1^ and 2850 cm^-1^ are evidence of the presence of long aliphatic chains, and the identified peaks correspond to vibrations of the –CH_3_ methyl group and the -CH_2_- methylene group, respectively (Dong et al., 2014). The presence of the –SH group in the BC spectrum is confirmed by the broad band at a wave length of ~2480cm^-1^. Towards the lower wave lengths, the spectra of the materials start to vary significantly. In the case of the control soil (S), the band at υ=1880 cm^-1^ may indicate the presence of the C=O group near the chlorine atom. In all three spectra, a band occurs in the wavenumber range of 1650-1600 cm^-1^, which evidences the presence of the C=O bond in amides or the C=C bond in the aromatic structures (Keiluweit et al., 2010). The next two peaks that occur only in the SL spectrum at the wavenumbers of 1575cm^-1^ and 1540cm^-1^ indicate the presence of NH_2_ groups in amines and NH groups in amides, respectively. The peak at υ=1467 cm^-1^ and 1415 cm^-1^, both in the S and BC spectra, reveals ring stretching in C=C (Cheng and Lehmann, 2009). A low intensity band at a wavenumber of 1258cm^-1^ in the BC spectrum confirms the presence of a nitro group. In all three spectra at a wavenumber of ~1030 cm^-1^, a narrow intense band is present, which is characteristic of C-O vibrations in the carboxyl group (Dong et al., 2014). The bands in the spectrum at υ=700-800 cm^-1^ indicate β–rings of pyridines (Das et al., 2009).

**XPS study of soil, sewage sludge and biochar**

In the composition of the control soil (S), the predominant elements are oxygen (50At%) in the form of C-O (66.3At%) and C=O (33.7At%) as well as silicon (26.4At%) exclusively as SiO_2_. Moreover, the content of carbon was determined (14At%), but its forms varied more than in the case of SL and BC. The forms C-C/C-H (49.2At%) and C-O-C/C-OH (31.6At%) dominate. As far as nitrogen forms are concerned, in most cases these are bonds N-C (60.2At%), N-C=O (28At%) and N-O (11.8At%).

In the input materials, i.e. SL and BC, Al and Fe were not found to be present (Table S3). Unlike SL, in BC potassium compounds in oxide form and sulfur compounds, also in oxide form, were determined. As regards the composition of both materials, carbon is the dominant element, but in the BC composition there are more oxygen bonds, both with a single C-O bond and with a double C=O bond as well as in the form of carbonates (3.3At%). In the sewage sludge, oxygen occurs only as C=O (72.2At%) and C-O (27.8At%), while in the case of BC, in turn, the content of both forms, C=O (48.2At%) and C-O (32.6At%), does not differ so drastically. In the case of BC, an important issue is the fact that a part of oxygen (19.2At%) occurs as H_2_O and adsorbed O_2_. This may indicate that the results for the adsorption of PTEs on BC will be underestimated due to blocking of adsorption sites by H_2_O and O_2_ molecules. The variation in nitrogen forms is smaller in the case of sewage sludge (SL), where it occurs mainly as N-C (77.4At%) and N-C=O (22.6At%), while in BC nitrogen also occurs as N-O and pyridine (Jansen and Vanbekkum, 1995). Moreover, a significant difference was found for silicon forms; SiO_2_ (66.3At%) and organosilicon (siloxanes) (33.7At%) dominate in BC, whereas in the case of SL silicon occurs only as siloxanes. Given that siloxane groups are characterized by greater sorption capacity, it can be concluded that their varying contents in the composition of SL and BC will affect the sorption capacity of both materials. Furthermore, phosphorus as phosphates was identified in the composition of both materials, in a larger amount in the case of SL (0.8At%).

**Effect of sewage sludge and/or biochar on soil properties**

The physicochemical properties of the native soil, SL and BC are presented and described in SI. In this section, the effect of SL or SL/BC mixture on the properties of the control soil is described. Adding SL to the soil caused a decrease in soil pH, CEC, DOC content, specific surface area (S_BET_), pore volume (V_p_), micropore area (S_micro_), and micropore volume (V_micro_) (Table 1). On the other hand, increased TOC and N_t_ content, hydrolytic acidity (H_h_) and pore size (PS) were found in the SL-amended soil compared to the control (un-amended) soil. The decrease in these parameters was attributable to their lower values in SL than in the soil. Likewise, the increase in the above-mentioned parameters was due to their higher values in SL than in the soil (Table 1).

FTIR spectrum analysis showed (Fig. S1) that adding of SL to the soil caused an increased intensity of the transmission bands at 1630 cm^-1^ and 770 cm^-1^, supporting the presence of, respectively, C=O groups in amides or C=C groups in the aromatic structures (Keiluweit et al., 2010) and β–rings of pyridines (Das et al., 2009), as well as a decreased intensity of the bands at 3430, 2920 and 2850 cm^-1^, supporting the presence of stretching vibrations of the –OH group (Yuan et al., 2011) and vibrations of the–CH_3_ and –CH_2_ groups (Dong et al., 2014).

Adding SL to the soil did not affect the qualitative composition of carbon and iron, but the proportions between the oxygen forms changed (Table S3). In the SL-amended soil, C-O started to dominate (76.9 At%), whereas the percentage of the C=O form decreased (23.1 At%). N-C=O groups were also characterized by a higher percentage in the SL-amended soil (59.7 At%). The presence of quaternary nitrogen, which was not observed in the control soil, was also found in the SL-amended soil (11.4 At%).

Adding sewage sludge mixed with biochar (BC/SL) to the soil did not affect significantly (except of few cases) its properties compared to the SL-amended soil (Table 1). Also, no significant relationships were observed between the rate of biochar added to sewage sludge and the changes in the properties of the amended soil. The lack of significant effect of BC on the physicochemical properties of the SL-amended soil was probably attributable to the low contribution of BC which was not able to exert a significant impact on physico-chemical properties of soil. No tendency between the biochar dosage and TOC content could be related to not equal distribution of the biochar in the control soil. Moreover, due to low mass of the BCs particles and the location of the field-experiment, where wind had an open border, BC could be extracted by wind, especially during the field cultivation. Furthermore, the loss of TOC could be also affected by the microbial activity and the decomposition of the organic matter.

Also, the addition of sewage sludge with biochar to the soil did not change significantly the location of the bands in the FTIR spectrum in comparison to the SL-amended soil. The intensity of some bands was however found to vary (Fig. S1). There was increased band intensity with increasing BC rate for the bands (1) 3430 cm^-1^, (2) 2920 cm^-1^ and (3) 2850 cm^-1^ as well as increased band intensity in the following order BC10<BC2.5<BC5.0 for the bands (4) 1880 cm^-1^, (5) 1630 cm^-1^, (6) 1030 cm^-1^ and (7) 770 cm^-1^. As regards most of the bands (1)-(5), the peak intensities for the treatments with BC addition were higher compared to the control soil amended with sewage sludge alone. In the case of the other bands, (6) and (7) as well as (5) for the treatment with the 10% BC rate, the intensities were lower than for the sewage sludge-amended soil. In the SL-amended soil with BC addition, the bands that were present exclusively in the SL and BC spectra were not found to be present (Fig. S1).

XPS analysis (Table S3) showed that after adding BC with SL to the soil, the carbon content increased for the treatments with the 2.5% and 5% BC rates as well as the nitrogen content for all SL/BC treatments, whereas the oxygen content decreased for the treatments with the 2.5% and 5% BC rates in comparison to the SL-amended soil. Moreover, it was found that new forms of nitrogen appeared, i.e. pyridine for S+SL+BC2.5 and S+SL+BC10, and that the N-C=O forms disappeared for the treatment with the 2.5% rate of biochar. The N-O content decreased for all treatments tested, but it is worth noting that the decrease in N-O content was proportional to the BC rate. On the other hand, the content of the N-C form increased in comparison to the S+SL treatment. A significant difference was noted for N-C=O; this form was not identified for the treatment S+SL+BC2.5, but in the case of the other treatments its content decreased compared to the SL-amended soil. In turn, Fe was partially oxidized after adding BC to SL and to the soil, from Fe(II) to Fe(III), and the increase in Fe(III) content was proportional to the BC rate. The silicon content decreased in the treatment S+SL+BC2.5, but did not change for S+SL+BC5.0 and increased in the treatment S+SL+BC10 compared to the soil with SL alone. A drastic decrease in Al content was also found, but only at the highest percentage of biochar in SL (S+SL+BC10).

**Table S1.** The basic parameters of heavy metal ions determination

| Metal | Lamp current [mA] |  | Wave length [nm] | Slit width [nm] | Acetylene flow [L/min] | Air flow [L/min] |
| --- | --- | --- | --- | --- | --- | --- |
| Cd | 4 |  | 228.8 | 0.5 | 2 | 13.5 |
| Cu | 4 |  | 324.8 | 0.5 | 2 | 13.5 |
| Ni | 4 |  | 232 | 0.2 | 2 | 13.5 |
| Zn | 5 |  | 213.9 | 1 | 2 | 13.5 |

**Table S2.** The content of the available forms of potassium, phosphorous and magnesium

| Sample | K_2_O  [mg/100g] | P_2_O_5_  [mg/100g] | MgO  [mg/100g] |
| --- | --- | --- | --- |
| Soil | 16.0 | 14.4 | 3.7 |
| S+SL | 15.9 | 16.7 | 3.9 |
| S+SL+BC2.5 | 14.5 | 13.3 | 3.8 |
| S+SL+BC5.0 | 20.3 | 15.5 | 3.6 |
| S+SL+BC10 | 18.5 | 14.4 | 3.9 |

**Table S3.** XPS data of studied materials

| Sample | Transition/  Subtotal (%At) | Peak energy (eV) | | At(%) | | | Chemical state |
| --- | --- | --- | --- | --- | --- | --- | --- |
| SL | C 1s  (82.7) | 284.7 | 86.5 | | | C-C/ C-H | |
|  |  | 286.1 | 7.6 | | | C-O-C/C-OH | |
|  |  | 287.6 | 2 | | | C=O | |
|  |  | 288.6 | 3.9 | | | O=C-O^-^ | |
|  | O 1s  (11.5) | 531.6 | 72.2 | | | C=O | |
|  |  | 532.8 | 27.8 | | | C-O | |
|  | N 1s  (1.5) | 399.7 | 77.4 | | | N-C | |
|  |  | 400.6 | 22.6 | | | N-C=O | |
|  | Si 2p  (1.3) | 102.4 | 100 | | | Organic Si | |
|  | Ca 2p  (2.1) | 347.2 | 100 | | | CaCO_3_ | |
|  | P 2p  (0.8) | 133.7 | 100 | | | Metal phosphate | |
| BC | C 1s  (80.5) | 284.7 | 68.8 | | | C-C/ C-H | |
|  |  | 286.1 | 17.1 | | | C-O-C/C-OH | |
|  |  | 287.4 | 5.2 | | | C=O | |
|  |  | 288.7 | 5.6 | | | O=C-O^-^ | |
|  |  | 209.5 | 3.3 | | | carbonates | |
|  | O 1s  (14) | 531.8 | 48.2 | | | C=O | |
|  |  | 533.1 | 32.6 | | | C-O | |
|  |  | 534.1 | 19.2 | | | H_2_O, O_2_ sorbed | |
|  | N 1s  (1.1) | 398.4 | 18.3 | | | pyridine | |
|  |  | 399.9 | 31 | | | N-C | |
|  |  | 401 | 42.3 | | | N-C=O | |
|  |  | 403 | 8.4 | | | N-O | |
|  | Si 2p  (0.7) | 102.1 | 33.7 | | | Organic Si | |
|  |  | 103.7 | 66.3 | | | SiO_2_ | |
|  | Ca 2p  (1.4) | 347.7 | 100 | | | Ca_3_(PO_4_)_2_ | |
|  | P 2p  (0.4) | 132.7 | 100 | | | Metal phosphate | |
|  | K 2p  (1.7) | 293.2 | 100 | | | K-O | |
|  | S 2p  (0.4) | 169.2 | 100 | | | Metal sulfate | |
| Soil | C 1s  (14) | 284.7 | 49.2 | | | C-C/ C-H | |
|  |  | 286.3 | 31.6 | | | C-O-C/C-OH | |
|  |  | 287.3 | 7.5 | | | C=O | |
|  |  | 288.5 | 11.7 | | | O=C-O^-^ | |
|  | O 1s  (50) | 531.7 | 33.7 | | | C=O | |
|  |  | 532.7 | 66.3 | | | C-O | |
|  | N 1s  (1.2) | 399.9 | 60.2 | | | N-C | |
|  |  | 400.9 | 28 | | | N-C=O | |
|  |  | 402.6 | 11.8 | | | N-O | |
|  | Mg 1s  (0.2) | 1301.7 | 100 | | | Mg metal | |
|  | Si 2p  (26.4) | 103.1 | 100 | | | SiO_2_ | |
|  | Ca 2p  (0.7) | 347.2 | 100 | | | CaCO_3_ | |
|  | K 2p  (0.6) | 292.2 | 100 | | | K-O | |
|  | Al 2p  (5.9) | 74.5 | 100 | | | Al_2_O_3_ | |
|  | Fe 2p  (1) | 709.3-716.4 | 15.6 | | | Fe(II) | |
|  |  | 712-719 | 84.4 | | | Fe(II) | |
| S+SL | C 1s  (12.7) | 284.7 | 50.6 | | | C-C/ C-H | |
|  |  | 286.2 | 30.6 | | | C-O-C/C-OH | |
|  |  | 287.3 | 9.4 | | | C=O | |
|  |  | 288.7 | 9.5 | | | O=C-O^-^ | |
|  | O 1s  (51.5) | 531.5 | 23.1 | | | C=O | |
|  |  | 532.7 | 76.9 | | | C-O | |
|  | N 1s  (0.9) | 399.2 | 17.8 | | | N-C | |
|  |  | 400.3 | 59.7 | | | N-C=O | |
|  |  | 401.4 | 11.4 | | | Quaternary N | |
|  |  | 402.8 | 11.1 | | | N-O | |
|  | Mg 1s  (0.3) | 1303.2 | 100 | | | Mg metal | |
|  | Si 2p  (27.6) | 103 | 100 | | | SiO_2_ | |
|  | Ca 2p  (0.5) | 351.7 | 100 | | | Ca_3_(PO_4_)_2_ | |
|  | K 2p  (0.7) | 293.3 | 100 | | | K-O | |
|  | Al 2p  (5.1) | 74.3 | 100 | | | Al_2_O_3_ | |
|  | Fe 2p  (0.8) | 709-715.5 | 16.1 | | | Fe(II) | |
|  |  | 712-719.4 | 83.9 | | | Fe(III) | |
| S+SL+BC2.5 | C 1s  (13.8) | 284.7 | 37.0 | | | C-C/ C-H | |
|  |  | 286.3 | 40.8 | | | C-O-C/C-OH | |
|  |  | 287.7 | 13.4 | | | C=O | |
|  |  | 288.9 | 8.90 | | | O=C-O^-^ | |
|  | O 1s  (51.1) | 531.3 | 26.5 | | | C=O | |
|  |  | 532.4 | 73.5 | | | C-O | |
|  | N 1s  (2.1) | 398.7 | 17.8 | | | pyridine | |
|  |  | 400.0 | 59.7 | | | N-C | |
|  |  | 401.3 | 11.4 | | | Quaternary N | |
|  |  | 402.8 | 11.1 | | | N-O | |
|  | Mg 1s  (0.2) | 1302.5 | 100 | | | Mg metal | |
|  | Si 2p  (24.5) | 103 | 100 | | | SiO_2_ | |
|  | Ca 2p  (0.6) | 348.5 | 100 | | | Ca_3_(PO_4_)_2_ | |
|  | K 2p  (0.9) | 294 | 100 | | | K-O | |
|  | Al 2p  (5.7) | 74.4 | 100 | | | Al_2_O_3_ | |
|  | Fe 2p  (1.0) | 709.3-715.8 | 15.2 | | | Fe(II) | |
|  |  | 711.8-721 | 84.8 | | | Fe(III) | |
| S+SL+BC5.0 | C 1s  (13.3) | 284.7 | 36.0 | | | C-C/ C-H | |
|  |  | 286.3 | 41.6 | | | C-O-C/C-OH | |
|  |  | 287.8 | 14.9 | | | C=O | |
|  |  | 288.9 | 7.60 | | | O=C-O^-^ | |
|  | O 1s  (50.8) | 531.5 | 22.2 | | | C=O | |
|  |  | 532.6 | 77.8 | | | C-O | |
|  | N 1s  (1.0) | 399.3 | 19.6 | | | N-C | |
|  |  | 400.2 | 55.9 | | | N-C=O | |
|  |  | 401.5 | 18.4 | | | Quaternary N | |
|  |  | 403 | 6.1 | | | N-O | |
|  | Mg 1s  (0.5) | 1303 | 100 | | | Mg metal | |
|  | Si 2p  (26.2) | 103 | 100 | | | SiO_2_ | |
|  | Ca 2p  (0.7) | 348.5 | 100 | | | Ca_3_(PO_4_)_2_ | |
|  | K 2p  (0.9) | 294.5 | 100 | | | K-O | |
|  | Al 2p  (6.0) | 74.5 | 100 | | | Al_2_O_3_ | |
|  | Fe 2p  (1.0) | 709.2-715.8 | 10.6 | | | Fe(II) | |
|  |  | 712-720.5 | 89.4 | | | Fe(III) | |
| S+SL+BC10 | C 1s  (11.2) | 284.7 | 43 | | | C-C/ C-H | |
|  |  | 286.2 | 36 | | | C-O-C/C-OH | |
|  |  | 287.8 | 15.6 | | | C=O | |
|  |  | 289 | 5.5 | | | O=C-O^-^ | |
|  | O 1s  (51.6) | 531.4 | 23.7 | | | C=O | |
|  |  | 532.7 | 76.3 | | | C-O | |
|  | N 1s  (1.5) | 398.4 | 6.1 | | | pyridine | |
|  |  | 399.6 | 36.7 | | | N-C | |
|  |  | 400.4 | 39.1 | | | N-C=O | |
|  |  | 401.3 | 13 | | | Quaternary N | |
|  |  | 402.6 | 5 | | | N-O | |
|  | Mg 1s  (0.3) | 1303.2 | 100 | | | Mg metal | |
|  | Si 2p  (28.8) | 102.9 | 100 | | | SiO_2_ | |
|  | Ca 2p  (0.6) | 351.7 | 100 | | | Ca_3_(PO_4_)_2_ | |
|  | K 2p  (0.8) | 293.2 | 100 | | | K-O | |
|  | Al 2p  (4.3) | 74.2 | 100 | | | Al_2_O_3_ | |
|  | Fe 2p  (0.8) | 708.6-715.5 | 10 | | | Fe(II) | |
|  |  | 711.8-719.5 | 90 | | | Fe(III) | |
|  |  |  |  | |  | | |

**Table S4. The content of metals in the soil and SL [mg/kg]**

| Sample | Zn | Cu | Cr | Ni | Cd | Pb | Co | Mn | Fe | Al |
| --- | --- | --- | --- | --- | --- | --- | --- | --- | --- | --- |
| Soil | 19.42 | 2.43 | 13.46 | 4.12 | 0.83 | 13.75 | 2.34 | 216.90 | 3947.43 | 904.62 |
| SL | 624.13 | 75.80 | 18.60 | 12.84 | 1.53 | 23.12 | 2.49 | 74.18 | 4506.76 | 854.59 |

**Table S5.** Selected properties of studied metals.

| Metal | Hydrated radius [Å] | Stability constants of complexes (pK) | | | | | | |  | |
| --- | --- | --- | --- | --- | --- | --- | --- | --- | --- | --- |
|  |  | S_2_O_3_^2-^ | SO_3_^2-^ | SO_4_^2-^ | P_2_O_7_^4-^ | P_3_O_9_^3-^ | P_4_O_12_^4-^ | P_3_O_10_^5-^ | |  |
| Cd | 4.26 | 3.9 | 4.2 | 2.2 | 8.7 | - | - | 9.8 | |  |
| Cu | 4.20 | 12.3 | - | 2.3 | 6.7 | - | 3.2 | 9.8 | |  |
| Ni | 4.04 | 2.1 | - | 2.3 | 5.8 | 3.2 | 4.95 | - | |  |
| Zn | 4.30 | 2.3 | - | 2.4 | 6.5 | - | - | 9.7 | |  |

**Table S6.** Parameters of PTE sorption isotherms fitted with Temkin model and sorption kinetics fitted to PFO and Elovich models.

| Metal | Sample | Isotherm model | | | Kinetics models | | | | | |  | | |
| --- | --- | --- | --- | --- | --- | --- | --- | --- | --- | --- | --- | --- | --- |
|  |  | Temkin | | | Pseudo –first order | | | Elovich | | | |  |  |
|  |  | B | K_T_ | R^2^ | k_1_(·10^-3^) | R^2^ | β | | α | R^2^ | | |  |
| Cd | S | 1.12 | 0.27 | 0.711 | 2.7 | 0.769 | 3.47 | | 1.16 | 0.920 | | |  |
|  | S+SL | 1.55 | 0.23 | 0.692 | 3.4 | 0.767 | 2.44 | | 1.44 | 0.946 | | |  |
|  | S+SL+BC2.5 | 1.99 | 0.17 | 0.875 | 3.4 | 0.624 | 2.44 | | 65.7 | 0.942 | | |  |
|  | S+SL+BC5.0 | 0.13 | 2.06 | 0.698 | 3.9 | 0.583 | 2.23 | | 16.1 | 0.821 | | |  |
|  | S+SL+BC10 | 0.95 | 0.28 | 0.886 | 1.6 | 0.242 | 3.92 | | 12.8 | 0.785 | | |  |
|  | SL | 7.09 | 1.96 | 0.933 | 3.3 | 0.804 | 0.49 | | 101 | 0.843 | | |  |
|  | BC | 1.54 | 4.9 | 0.922 | 3.3 | 0.769 | 1.38 | | 1.66 | 0.948 | | |  |
| Cu | S | 0.47 | 2.0 | 0.911 | 4.0 | 0.593 | 2.81 | | 0.71 | 0.940 | | |  |
|  | S+SL | 0.82 | 0.31 | 0.839 | 3.7 | 0.699 | 2.83 | | 0.70 | 0.970 | | |  |
|  | S+SL+BC2.5 | 0.88 | 0.24 | 0.761 | 4.8 | 0.676 | 3.22 | | 1.13 | 0.887 | | |  |
|  | S+SL+BC5.0 | 0.87 | 0.26 | 0.798 | 3.4 | 0.590 | 3.39 | | 2.43 | 0.867 | | |  |
|  | S+SL+BC10 | 0.72 | 0.31 | 0.784 | 3.3 | 0.407 | 4.09 | | 2.55 | 0.882 | | |  |
|  | SL | 4.86 | 1.04 | 0.913 | 4.1 | 0.770 | 0.39 | | 11.0 | 0.953 | | |  |
|  | BC | 1.61 | 6.02 | 0.692 | 3.4 | 0.563 | 1.78 | | 1.54 | 0.890 | | |  |
| Ni | S | 1.21 | 0.47 | 0.909 | 3.6 | 0.549 | 2.49 | | 197.3 | 0.933 | | |  |
|  | S+SL | 1.73 | 0.15 | 0.862 | 3.7 | 0.575 | 1.49 | | 4.88 | 0.924 | | |  |
|  | S+SL+BC2.5 | 1.77 | 0.13 | 0.808 | 4.7 | 0.443 | 1.60 | | 6.32 | 0.865 | | |  |
|  | S+SL+BC5.0 | 1.47 | 0.13 | 0.849 | 4.0 | 0.423 | 1.74 | | 0.80 | 0.889 | | |  |
|  | S+SL+BC10 | 1.27 | 0.14 | 0.846 | 3.6 | 0.438 | 2.01 | | 5.27 | 0.868 | | |  |
|  | SL | 3.14 | 0.74 | 0.969 | 3.0 | 0.646 | 0.76 | | 22.8 | 0.943 | | |  |
|  | BC | 1.70 | 0.53 | 0.870 | 4.0 | 0.497 | 1.19 | | 30.1 | 0.937 | | |  |
| Zn | S | 0.43 | 0.65 | 0.772 | 3.0 | 0.805 | 7.81 | | 5.50 | 0.945 | | |  |
|  | S+SL | 0.70 | 0.23 | 0.832 | 3.1 | 0.769 | 4.05 | | 0.76 | 0.983 | | |  |
|  | S+SL+BC2.5 | 0.91 | 0.21 | 0.797 | 3.1 | 0.580 | 4.21 | | 14.0 | 0.883 | | |  |
|  | S+SL+BC5.0 | 0.86 | 0.21 | 0.863 | 2.9 | 0.606 | 2.71 | | 3.07 | 0.878 | | |  |
|  | S+SL+BC10 | 0.74 | 0.17 | 0.920 | 2.9 | 0.586 | 2.80 | | 2.56 | 0.865 | | |  |
|  | SL | 3.82 | 2.56 | 0.913 | 3.4 | 0.672 | 0.55 | | 18.3 | 0.954 | | |  |
|  | BC | 0.87 | 1.37 | 0.903 | 2.9 | 0.664 | 1.26 | | 2.88 | 0.952 | | |  |

*B-* the Temkin constant related to adsorption heat*, K_T_* -the constant of equilibrium binding [L/g] related to the maximum binding energy, R – regression coefficient, k_1_ [1/min] and k_2_ [g/mg·min] - the rate constants of the pseudo – first – order equation and pseudo – second – order equation, β- Elovich constant [g/mg], α- initial adsorption rate [mg/(g·min)].

**Table S7.** Correlation coefficients between sorption capacity obtained experimentally (a_exp,i_) and physico-chemical properties of studied treatments

|  | Cd | Cu | Ni | Zn |
| --- | --- | --- | --- | --- |
| pH | -0.785 | -0.987 | -0.312 | -0.936 |
| H_h_ | 0.892 | 0.977 | 0.363 | 0.989 |
| CEC | -0.878 | -0.855 | -0.654 | -0.703 |
| TOC | 0.638 | 0.700 | 0.173 | 0.509 |
| DOC | 0.422 | -0.066 | 0.748 | -0.073 |
| N | 0.674 | 0.797 | 0.154 | 0.741 |
| Ash | -0.691 | -0.802 | -0.450 | -0.883 |
| S_BET_ | -0.612 | -0.944 | -0.215 | -0.995 |
| V_p_ | -0.147 | -0.594 | 0.217 | -0.562 |
| PS | 0.623 | 0.695 | 0.472 | 0.803 |
| S_micro_ | -0.057 | 0.037 | 0.242 | 0.133 |
| V_micro_ | -0.021 | -0.063 | 0.377 | 0.038 |

Red color menas means statistically significent (P ≥ 0.05)

**Table S8.** Correlation coefficients between K, n parameters based on FM and the physico-chemical properties of treatments

| Properties |  |  | K |  |  |  | *n* |  |
| --- | --- | --- | --- | --- | --- | --- | --- | --- |
|  | Cd | Cu | Ni | Zn | Cd | Cu | Ni | Zn |
| pH | -0.966 | 0.038 | 0.080 | 0.109 | 0.627 | 0.089 | 0.086 | -0.046 |
| H_h_ | 0.939 | -0.015 | -0.173 | -0.249 | -0.738 | -0.006 | 0.060 | 0.155 |
| CEC | -0.639 | -0.392 | -0.437 | -0.308 | 0.773 | 0.402 | 0.446 | 0.382 |
| TOC | 0.695 | 0.003 | 0.237 | 0.308 | -0.228 | -0.313 | -0.455 | -0.288 |
| DOC | -0.219 | 0.797 | 0.445 | 0.429 | -0.569 | -0.668 | -0.436 | -0.524 |
| N | 0.894 | -0.103 | -0.143 | -0.035 | -0.376 | -0.177 | -0.159 | 0.009 |
| Ash | -0.707 | -0.168 | 0.195 | 0.278 | 0.823 | 0.071 | -0.123 | -0.150 |
| S_BET_ | -0.922 | 0.177 | 0.316 | 0.424 | 0.654 | -0.206 | -0.252 | -0.336 |
| V_p_ | -0.575 | 0.455 | 0.210 | 0.325 | 0.079 | -0.434 | -0.253 | -0.339 |
| PS | 0.650 | 0.163 | -0.227 | -0.314 | -0.803 | -0.041 | 0.170 | 0.183 |
| S_micro_ | -0.179 | 0.150 | 0.025 | -0.256 | -0.383 | 0.270 | 0.317 | 0.174 |
| V_micro_ | -0.293 | 0.327 | 0.112 | -0.150 | -0.449 | 0.111 | 0.219 | 0.053 |

Red color menas means statistically significent (P ≥ 0.05)

**Table S9.** Correlation coefficients between maximal desorption of heavy metal ions and the physico-chemical properties of treatments

| Properties | Cd | Cu | Ni | Zn |
| --- | --- | --- | --- | --- |
| pH | 0.531 | 0.806 | 0.933 | 0.884 |
| H_h_ | -0.564 | -0.768 | -0.811 | -0.930 |
| CEC | -0.035 | 0.379 | 0.683 | 0.505 |
| TOC | -0.231 | -0.582 | -0.898 | -0.464 |
| DOC | -0.085 | 0.515 | 0.167 | 0.184 |
| N | 0.763 | 0.767 | -0.968 | -0.778 |
| Ash | 0.624 | 0.494 | 0.521 | 0.791 |
| S_BET_ | 0.510 | 0.821 | 0.723 | 0.954 |
| V_p_ | -0.088 | 0.671 | 0.464 | 0.527 |
| PS | -0.620 | -0.447 | -0.444 | -0.756 |
| S_micro_ | 0.506 | 0.204 | 0.462 | 0.014 |
| V_micro_ | 0.467 | 0.374 | 0.551 | 0.116 |

Red color menas means statistically significent (P ≥ 0.05)


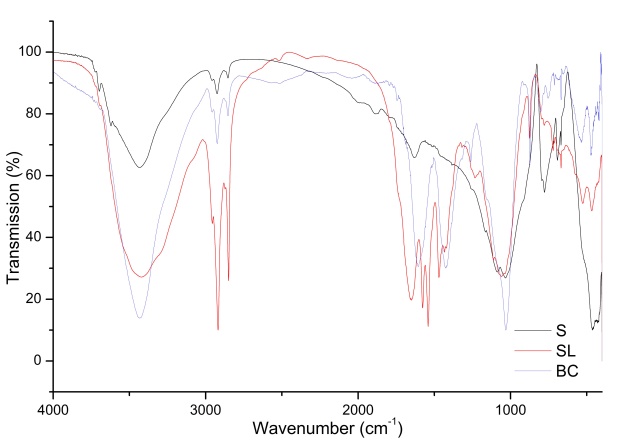

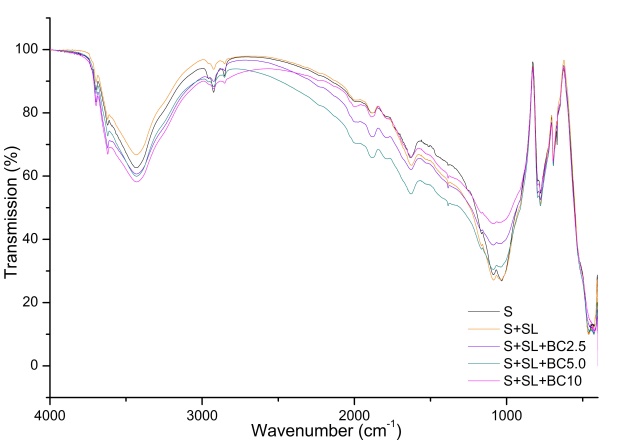


**Fig. S1.** FT-IR/PAS spectra of starting materials S, SL, BC and mixed of S, SL, BC compering to reference soil (S).

**REFERENCES**

Cheng, C.-H., Lehmann, J., 2009. Ageing of black carbon along a temperature gradient. Chemosphere 75, 1021–1027. doi:10.1016/j.chemosphere.2009.01.045

Das, D.D., Schnitzer, M.I., Monreal, C.M., Mayer, P., 2009. Chemical composition of acid–base fractions separated from biooil derived by fast pyrolysis of chicken manure. Bioresour. Technol. 100, 6524–6532. doi:10.1016/j.biortech.2009.06.104

Dong, X., Wang, C., Li, H., Wu, M., Liao, S., Zhang, D., Pan, B., 2014. The sorption of heavy metals on thermally treated sediments with high organic matter content. Bioresour. Technol. 160, 123–128. doi:10.1016/j.biortech.2014.01.006

Jansen, R., Vanbekkum, H., 1995. Xps of Nitrogen-Containing Functional-Groups on Activated Carbon. Carbon 33, 1021–1027. doi:10.1016/0008-6223(95)00030-H

Keiluweit, M., Nico, P.S., Johnson, M.G., Kleber, M., 2010. Dynamic Molecular Structure of Plant Biomass-Derived Black Carbon (Biochar). Environ. Sci. Technol. 44, 1247–1253. doi:10.1021/es9031419

Yuan, J.-H., Xu, R.-K., Zhang, H., 2011. The forms of alkalis in the biochar produced from crop residues at different temperatures. Bioresour. Technol. 102, 3488–3497. doi:10.1016/j.biortech.2010.11.018
